# Identification of Durum Wheat Cultivars and Their Tetraploid Relatives with Low Cadmium Content

**DOI:** 10.17113/ftb.58.01.20.6531

**Published:** 2020-03

**Authors:** Mehmet Tekin, Ahmet Cat, Sahriye Sönmez, Taner Akar

**Affiliations:** 1 Akdeniz University, Faculty of Agriculture, Department of Field Crops, 07059 Antalya, Turkey; 2 Akdeniz University, Faculty of Agriculture, Department of Plant Protection, 07059 Antalya, Turkey; 3 Akdeniz University, Faculty of Agriculture, Department of Soil Science, 07059 Antalya, Turkey

**Keywords:** durum wheat characterization, low Cd content, tetraploid wheat, marker assisted breeding

## Abstract

In this study, 71 durum wheat cultivars (*Triticum durum* Desf.), 22 emmer wheat (*Triticum dicoccum* L.) and 11 wild emmer (*Triticum dicoccoides* L.) genotypes were genetically characterized to determine the alleles associated with high cadmium (Cd) content. After genotypic characterization, 14 cultivars selected among all genotypes with low and high Cd content were phenotyped by a pot experiment to verify the genotypic data. Identification of 32 durum wheat, one emmer wheat and four wild emmer genotypes showed that they have alleles associated with high Cd content, while 68 genotypes of which 39 durum wheat, 21 emmer wheat and 7 wild emmer cultivars had alleles associated with low Cd content, respectively. Moreover, phenotypic data obtained from pot experiment were similar to the molecular data. To sum up, the marker successfully classified durum wheat cultivars into either high or low accumulators and these results can be safely used in breeding programs to improve new durum wheat cultivars with alleles associated with low Cd content. Due to routine use of phosphorus fertilizers in agricultural areas and other anthropogenic factors related to Cd toxicity, new durum wheat cultivars with low Cd content should be urgently developed for safe production of macaroni or other types of wheat products for human and animal consumption.

## INTRODUCTION

The accumulation of heavy metals in the soil has a major impact on the environment. Heavy metals accumulated in the soil are stored in plant tissues over time and, therefore, pose a threat to human and animal health (*1*, *2*). Cadmium (Cd) is one of the toxic metals which causes very serious health problem for humans. So far, diseases caused by Cd such as Itai-itai have been diagnosed (*2*). Metal industry, fossil fuels, domestic waste, application of pesticide and phosphorus fertilizers are the greatest sources of Cd pollution (*3*). Moreover, 30 000 t of Cd are added to environment annually and 13 000 t of these are caused by human activities (*4*). Many countries have determined several restrictions for the mass fraction of Cd in phosphorus fertilizers. Germany for instance has limited the Cd mass fraction in phosphorus fertilizers to 200 mg/kg (*5*). In Sweden, phosphorus fertilizers have been subjected to taxes when the Cd mass fraction exceeds 5 mg/kg and imports of phosphorous fertilizers with a Cd mass fraction above 100 mg/kg are prohibited (*6*). This implementation encourages the production of low-Cd fertilizers and reduces Cd input into the soil.

Cd is mostly accumulated in the human body through food consumption, mainly thanks to a high intake of cereals. It is known that the contribution of cereal products to daily Cd intake ranges from 20 to 43% (*7*). Wheat (*Triticum* L.) is the first among the cultivated plants in terms of production and harvested area worldwide and wheat products provide 20% of the daily calories and also 20% of the protein, especially in 94 developing countries with more than 4.5 billion people (*8*). There is a large genetic variation especially in durum wheat (*Triticum durum* Desf.) regarding Cd accumulation in the grain and it is known that this species has higher Cd content than other cool season cereals: rye<barley<oat<bread wheat<durum wheat (*6*-*9*). Food and Agriculture Organization of the United Nations (FAO) and The International Codex Alimentarius Commission of World Health Organization (WHO) have standardized the maximum allowable Cd mass fraction in wheat grain to be 0.1 mg/kg (*10*). In order to reduce Cd uptake and toxicity, many alternative methods such as the use of plant growth regulators and plant nutrients are applied (*2*). However, the most environmentally friendly and efficient strategy are the development of new genetic materials with low cadmium content (*2*) and breeding programs have been carried out in many countries for this purpose. For instance, in Canada, where Cd is one of the most serious environmental problems, durum wheat cultivars have been developed since 2003 *via* marker-assisted selection (MAS) (*11*-*13*). A major gene *Cdu1* (*14*), which is located in the long arm of chromosome 5B, controls the Cd accumulation in durum wheat (*15*, *16*). Several tightly linked markers with gene *Cdu1* have been developed to detect the allele associated with low content of Cd in durum wheat such as dominant SCAR marker *ScOPC20* (*15*) and co-dominant CAPS marker *usw47* (*17*). These markers can be used to characterize Cd accumulation in both tetraploid durum wheat and other tetraploid wheat relatives such as emmer (*Triticum dicoccum* L.) and wild emmer (*Triticum dicoccoides* L.).

The aim of the study was to molecularly characterize Turkish durum wheat gene pool and some emmer wheat and wild emmer genotypes *via usw47* marker for allele associated with low Cd content. Additionally, some selected durum wheat cultivars with alleles associated with low or high Cd content were tested in pot experiment to verify the molecular data.

## MATERIALS AND METHODS

### Genetic materials

Seventy-one durum wheat (*Triticum durum* Desf.) cultivars and advanced breeding lines with 2 universal controls, 24 emmer wheat (*Triticum dicoccum* L.) genotypes and 11 wild emmer (*Triticum dicoccoides* L.) genotypes (Table 1) were used as genetic materials in this study. Canadian durum wheat cultivar “Commander” and advanced line “Dt 812” kindly provided by Dr Y. Ruan from Agriculture and Agri-Food Canada were used as negative and positive control, respectively. Ten emmer wheat and all wild emmer genotypes were obtained from Turkish Seed Gene Bank (Ankara, Turkey). Other emmer wheat lines used in the study were developed *via* selection breeding in a project funded by TUBITAK (The Scientific and Technological Research Council of Turkey, Project No: 214O401).

**Table 1:** Different tetraploid germplasm materials used in the study

No.	Species	Genotype	No.	Species	Genotype	No.	Species	Genotype
1	*Triticum durum* Desf.	Zenit	37	*Triticum durum* Desf.	Tunca 79	73	*Triticum durum* Desf.	Dt 812
2	*Triticum durum* Desf.	Svevo	38	*Triticum durum* Desf.	Ankara 98	74	*Triticum dicoccum* L.	TR79489
3	*Triticum durum* Desf.	Saragolla	39	*Triticum durum* Desf.	Sis gd 14 v	75	*Triticum dicoccum* L.	TR61225
4	*Triticum durum* Desf.	Claudio	40	*Triticum durum* Desf.	Sis gd 14 e	76	*Triticum dicoccum* L.	TR69596
5	*Triticum durum* Desf.	Aydın 93	41	*Triticum durum* Desf.	Sis gd 14 c	77	*Triticum dicoccum* L.	TR69623
6	*Triticum durum* Desf.	Ege 88	42	*Triticum durum* Desf.	5	78	*Triticum dicoccum* L.	TR68784
7	*Triticum durum* Desf.	Fırat 93	43	*Triticum durum* Desf.	6	79	*Triticum dicoccum* L.	TR68789
8	*Triticum durum* Desf.	Fuatbey 2000	44	*Triticum durum* Desf.	12	80	*Triticum dicoccum* L.	TR68817
9	*Triticum durum* Desf.	Gap	45	*Triticum durum* Desf.	14	81	*Triticum dicoccum* L.	TR68857
10	*Triticum durum* Desf.	Gediz 75	46	*Triticum durum* Desf.	22	82	*Triticum dicoccum* L.	TR69632
11	*Triticum durum* Desf.	Harran 95	47	*Triticum durum* Desf.	29	83	*Triticum dicoccum* L.	TR72183
12	*Triticum durum* Desf.	Sarıçanak 98	48	*Triticum durum* Desf.	47	84	*Triticum dicoccum* L.	Advanced line 5
13	*Triticum durum* Desf.	Şölen 2002	49	*Triticum durum* Desf.	50	85	*Triticum dicoccum* L.	Advanced line 6
14	*Triticum durum* Desf.	Turabi	50	*Triticum durum* Desf.	57	86	*Triticum dicoccum* L.	Advanced line 11
15	*Triticum durum* Desf.	Tüten 2002	51	*Triticum durum* Desf.	69	87	*Triticum dicoccum* L.	Advanced line 13
16	*Triticum durum* Desf.	Diyarbakır 81	52	*Triticum durum* Desf.	71	88	*Triticum dicoccum* L.	Advanced line 14
17	*Triticum durum* Desf.	Altıntaş 95	53	*Triticum durum* Desf.	72	89	*Triticum dicoccum* L.	Advanced line 18
18	*Triticum durum* Desf.	Amanos 97	54	*Triticum durum* Desf.	75	90	*Triticum dicoccum* L.	Advanced line 24
19	*Triticum durum* Desf.	Maestrale	55	*Triticum durum* Desf.	101	91	*Triticum dicoccum* L.	Advanced line 35
20	*Triticum durum* Desf.	Aurea	56	*Triticum durum* Desf.	102	92	*Triticum dicoccum* L.	Advanced line 42
21	*Triticum durum* Desf.	Normanno	57	*Triticum durum* Desf.	103	93	*Triticum dicoccum* L.	Advanced line 50
22	*Triticum durum* Desf.	Gracale	58	*Triticum durum* Desf.	106	94	*Triticum dicoccum* L.	Advanced line 52
23	*Triticum durum* Desf.	Levante	59	*Triticum durum* Desf.	107	95	*Triticum dicoccum* L.	Advanced line 53
24	*Triticum durum* Desf.	Kunduru 1149	60	*Triticum durum* Desf.	110	96	*Triticum dicoccum* L.	Advanced line 57
25	*Triticum durum* Desf.	Eminbey	61	*Triticum durum* Desf.	111	97	*Triticum dicoccum* L.	Advanced line 60
26	*Triticum durum* Desf.	Kamut	62	*Triticum durum* Desf.	Dww tk 4	98	*Triticum dicoccoides* L	TGB 00777
27	*Triticum durum* Desf.	Çeşit 1252	63	*Triticum durum* Desf.	Dww tk 11	99	*Triticum dicoccoides* L	TGB 00791
28	*Triticum durum* Desf.	Dumlupınar	64	*Triticum durum* Desf.	Dww tk 12	100	*Triticum dicoccoides* L	TGB 000792
29	*Triticum durum* Desf.	Kızıltan 91	65	*Triticum durum* Desf.	Dww tk 14	101	*Triticum dicoccoides* L	TGB 045861
30	*Triticum durum* Desf.	Meram 2002	66	*Triticum durum* Desf.	Dww tk 15	102	*Triticum dicoccoides* L	TGB 045871
31	*Triticum durum* Desf.	Mirzabey 2000	67	*Triticum durum* Desf.	Kocasarı 2-3	103	*Triticum dicoccoides* L.	TGB 045910
31	*Triticum durum* Desf.	Yelken	68	*Triticum durum* Desf.	Kocasarı 4-1	104	*Triticum dicoccoides* L	TGB 045911
33	*Triticum durum* Desf.	Selçuklu 97	69	*Triticum durum* Desf.	Kocasarı 8-2	105	*Triticum dicoccoides* L	TGB 045912
34	*Triticum durum* Desf.	Altın 40/98	70	*Triticum durum* Desf.	Kocasarı 9-1	106	*Triticum dicoccoides* L	TGB 045913
35	*Triticum durum* Desf.	Kunduru414/44	71	*Triticum durum* Desf.	Kocasarı 16-1	107	*Triticum dicoccoides* L	TGB 045920
36	*Triticum durum* Desf.	Yılmaz 98	72	*Triticum durum* Desf.	Commander	108	*Triticum dicoccoides* L	TGB 038501

74-83 emmer and 98-108 wild emmer genotypes were obtained from Turkish Seed Gene Bank (Ankara, Turkey), 84-97 emmer genotypes were developed in a project funded by TUBITAK (The Scientific and Technological Research Council of Turkey, project no. 214O401)

### Genetic characterization

Seeds of all genotypes were sown on trays and then leaf samples were collected from plants for DNA extraction at 2-3 leaf stage. DNA was extracted according to cetyl trimethylammonium bromide (CTAB) method (*18*). The extracted DNA samples were loaded on agarose gel (Biomax, Thomas Scientific, Swedesboro, NJ, USA) with a DNA standard in order to determine the quality and concentration of the DNA and then were stored in sterile distilled water at -20 °C until use. To amplify *Cdu1* gene alleles by PCR, the co-dominant CAPS marker *usw47*, which was derived from an expressed sequence tag (EST) XBF474090 co-segregating with *Cdu1* (*16*), was used.

PCR was carried out as follows: the total volume of the reaction mixture was 15 μL containing 100 ng genomic DNA, 1× PCR buffer (Sigma Aldrich, Merck, St. Louis, MO, USA), 1.5 mM MgCI_2_ (Sigma Aldrich, Merck), 0.2 mM of dNTP mix (Thermo Fisher Scientific, Waltham, MA, USA) 0.4 μM of *usw47* forward primer (5’-GCTAGGACTTGATTCATTGAT-3’), 0.4 μM of *usw47* reverse primer (5’-AGTGATCTAAACGTTCTTATA-3’), 1.25 U Taq DNA polymerase (Sigma Aldrich, Merck). Amplification was performed in a thermocycler (MyGenie^TM^ 96; Bioneer, Daejon, Korea) under the following conditions: 94 °C initial denaturation for 5 min, 30 cycles at 94 °C for 30 s, annealing temperature 55 °C for 30 s, 72 °C for 1 min, and then a final extension of 10 min at 72 °C.

The PCR products were digested by *Hpy188I* (New England Biolabs, Ipswich, MA, USA) restriction enzyme after amplification. The total volume of the reaction mixture for enzymatic digestion was 15 μL containing 4 μL PCR product, 0.25 μL *Hpy188I* gene from *Helicobacter pylori*, 1× NEBuffer 4 (New England Biolabs) and 9.25 μL distilled water. Enzymatic digestion was performed in a thermo-shaker (Biosan, Riga, Latvia) under the following conditions: 37 °C for 1 h, 65 °C for 20 min and holding at 10 °C for 5 min and then the products were loaded in 2% agarose gel and visualized under UV light after staining with ethidium bromide (Sigma Aldrich, Merck).

### Pot experiment and elemental analysis

After molecular analysis, a small set of commonly cultivated 14 genotypes (Ege-88, Amanos-97, Sarıçanak 98, Şölen 2002, Turabi, Svevo, Zenit, Fırat-93, Fuatbey 2000, GAP, Gediz 75, Tüten 2002, Diyarbakır and Levante) was grown in pots in three replicates. The soil was mixed with acidic peat, in 1:1 ratio to increase the Cd uptake by plants, and then each pot was filled in with 2 kg of the mixture. A volume of 10 mL of CdCI_2_·H_2_O (Merck, Darmstadt, Germany) was added to each pot with automatic pipette to achieve final Cd mass fraction of 8 mg/kg. At physiologically ripening stage based on Zadoks growth scale (Z 98), grain and stem parts were sampled for each genotype and the samples were dried at 70 °C to constant mass. Dried plant samples of 0.5 g each were digested with 10 mL HNO_3_/HClO_4_ acid (4:1; Merck) mixture on a hotplate. The samples were then heated until a clear solution was obtained. The same procedure was repeated several times. The samples were filtered and diluted to 100 mL using distilled water, and then Cd mass fraction of the combusted samples with other elements such as P, Mg, Ca, K, Zn, Cu, Fe and Mn was measured by inductively coupled plasma-optical emission spectrometer (ICP-OES) (Optima, PerkinElmer Inc., Waltham, MA, USA). Additionally, soil in each pot was analyzed to determine total Cd accumulation from the soil in the biomass of each genotype at the end of the pot experiment.

### Statistical analysis

Basic statistical parameters such as mean and standard error of mean were determined. Analysis of variance (ANOVA) was performed with least significant difference (LSD) test at the 95% confidence level using SAS statistical software (*19*). Additionally, correlation and principal component analyses (PCA) were performed to determine relationships among the elements by XLSTAT statistical software (*20*).

## RESULTS AND DISCUSSION

### Genetic characterization

**Fig. 1:**
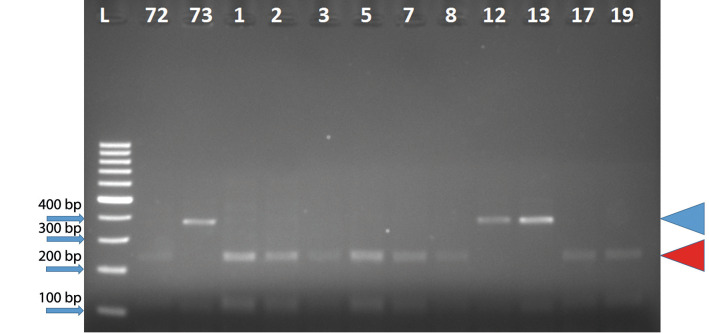
Results of PCR analysis of durum wheat alleles associated with the accumulation of Cd obtained from *usw47* marker. Red and blue symbols illustrate alleles associated with high and low content of Cd, respectively

**Fig. S1:**
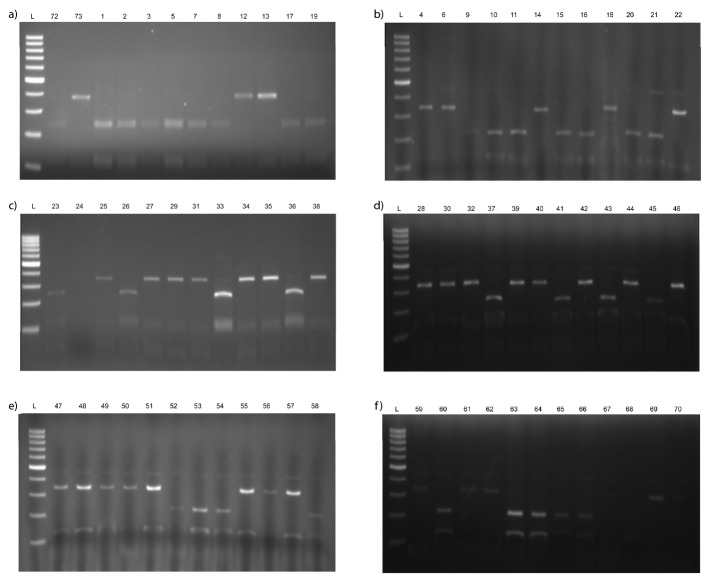
Results of PCR analysis of alleles of: a-c) durum wheat cultivars, and d-f) lines associated with the accumulation of Cd obtained from *usw47* marker

**Fig. S2:**
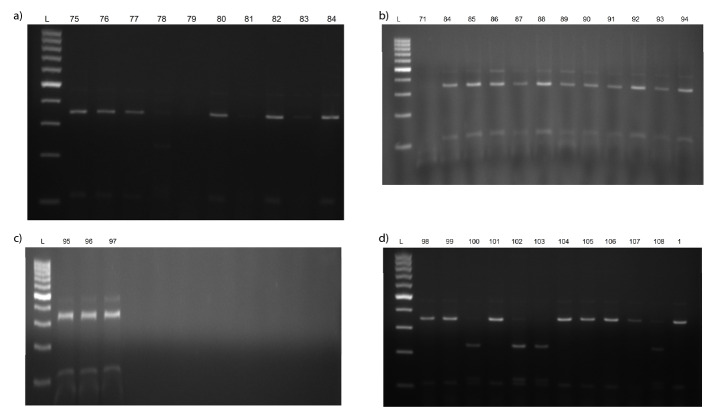
Results of PCR analysis of alleles of: a-c) emmer and d) wild emmer genotypes associated with the accumulation of Cd obtained from *usw47* marker

Fig. 1 shows the results of PCR analysis of alleles associated with the accumulation of Cd obtained from *usw47* marker. According to banding patterns of *usw47*, there are three possible alleles: for low Cd content, high Cd content and heterogeneous. Fig. S1 and Fig. S2 show all gel visualizations obtained from molecular analysis. Genotyping results of 108 tested tetraploid wheats are shown in Table 2.

**Table 2:** Determination of alleles of durum wheat cultivars associated with low and high Cd content based on molecular analysis

No.	Molecular evaluation	No.	Molecular evaluation	No.	Molecular evaluation
1	High	37	High	73	Low
2	High	38	Low	74	Low
3	High	39	Low	75	Low
4	Low	40	Low	76	Low
5	High	41	High	77	Low
6	Low	42	Low	78	High
7	High	43	High	79	Low
8	High	44	Low	80	Low
9	High	45	High	81	Low
10	High	46	Low	82	Low
11	High	47	Low	83	Low
12	Low	48	Low	84	Low
13	Low	49	Low	85	Low
14	Low	50	Low	86	Low
15	High	51	Low	87	Low
16	High	52	High	88	Low
17	High	53	High	89	Low
18	Low	54	High	90	Low
19	High	55	Low	91	Low
20	High	56	Low	92	Low
21	High	57	Low	93	Low
22	Low	58	High	94	Low
23	High	59	Low	95	Low
24	Low	60	High	96	Low
25	Low	61	Low	97	Low
26	High	62	Low	98	Low
27	Low	63	High	99	Low
28	Low	64	High	100	High
29	Low	65	High	101	Low
30	Low	66	High	102	High
31	Low	67	Low	103	High
31	Low	68	Low	104	Low
33	High	69	Low	105	Low
34	Low	70	Low	106	Low
35	Low	71	Low	107	Low
36	High	72	High	108	High

Based on the molecular analysis, 21 (52.5%) out of 40 durum wheat cultivars had alleles associated with high and 12 (47.5%) with low Cd content, and 19 (36.4%) out of 33 advanced breeding lines had alleles associated with high and 21 (63.6%) with low Cd content (Table 2 and Fig. S1). Additionally, only 1 (4%) of the 24 emmer wheat genotypes had alleles associated with high Cd content, and 7 (63.6%) of 11 wild emmer genotypes with low and 4 (36.4%) with high Cd content (Table 2 and Fig. S2). Similar results were obtained by Zimmerl *et al.* (*17*), who reported 166 (53%) of 314 tetraploid wheat genotypes associated with low Cd content and *usw47* marker can be successfully used to determine low Cd accumulators in tetraploid wheat accessions. Moreover, Vergine *et al.* (*21*) also genetically characterized tetraploid genotypes by a sequence-characterized amplified region (SCAR) marker, *ScOPC20* in terms of Cd accumulation. However, this marker allows to display two different alleles: one associated with low Cd content (band absent) and another with high Cd content (band present), therefore, heterogeneous state cannot be detected.

### Elemental analysis based on pot experiment

**Fig. 2:**
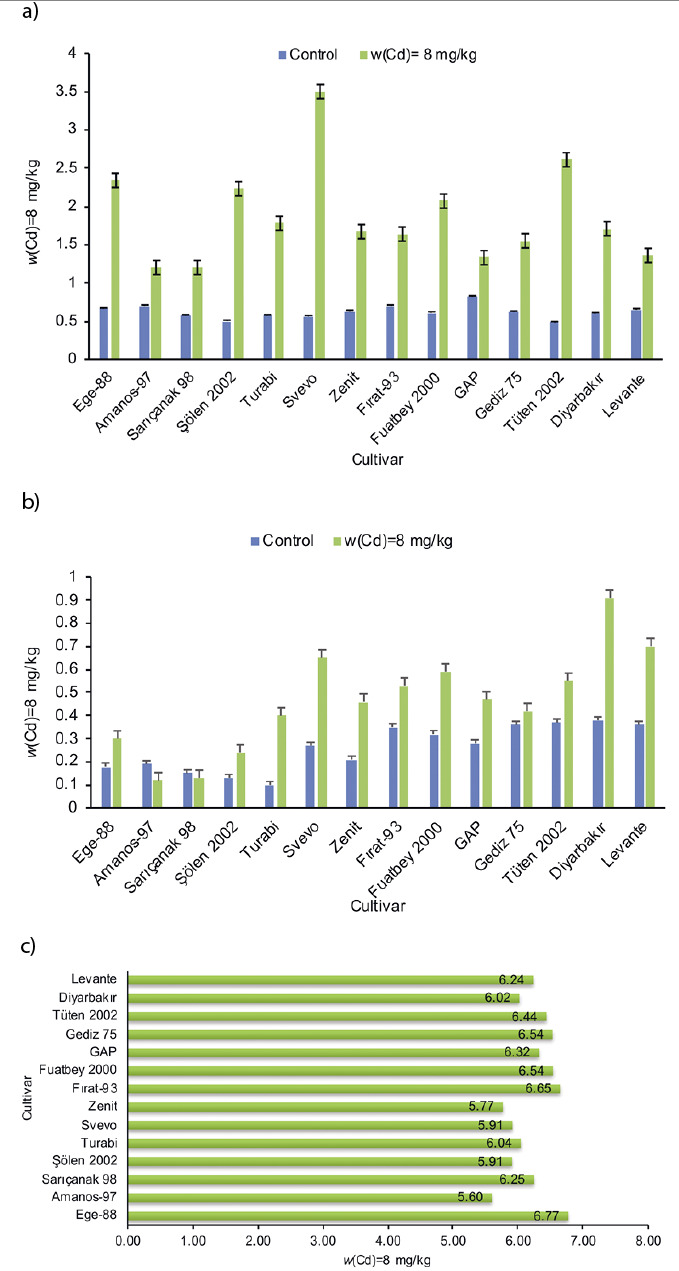
Mass fractions of Cd in: a) grain, b) stem and c) underground parts of durum wheat cultivars in both control and samples grown in the soil with the addition of *w*(Cd)=8 mg/kg

Fig. 2 shows Cd mass fractions in the grain, stem and underground parts of fourteen durum wheat cultivars. The results in Fig. 2a demonstrate that Cd addition (8 mg/kg) to the soil mixture clearly increases Cd accumulation in the grain. The cultivar Diyarbakır was the highest accumulator of Cd in the grain in both control and samples with added Cd (0.38 and 0.91 mg/kg respectively, Fig. 2a), while the control sample of Turabi cultivar and the sample of Amanos-97 cultivar grown in the soil with added Cd had the lowest Cd content in grain (0.1 and 0.12 mg/kg respectively, Table S1). All cultivars used in pot experiment except Amanos-97 and Sarıçanak 98 accumulated more Cd in grains after the addition of Cd to the soil (Table S1). In addition to cadmium, phosphorus, potassium, calcium, magnesium, iron, zinc, copper and manganese mass fractions were also determined (Table S1). A two-way ANOVA showed a significant difference (p<0.01) in the mass fractions of these elements among cultivars. Moreover, taking into account cultivar × treatment interaction, a significant difference was found in the mass fractions of all elements at the p=0.01 except for phosphorus (p<0.05). As expected, phenotypic data obtained from pot experiment for Cd accumulation in the grain were similar to molecular data. Low mass fraction of Cd was found in cultivars Ege-88, Amanos-97, Sarıçanak 98, Sölen 2002 and Turabi, which have the allele associated with low Cd content (Fig. 2a). Zimmerl *et al.* (*17*) and Perrier *et al.* (*22*) reported that varieties with the allele associated with high Cd content had 2.4-fold more Cd in the grain than the varieties with the allele associated with low Cd content.

**Fig. 3:**
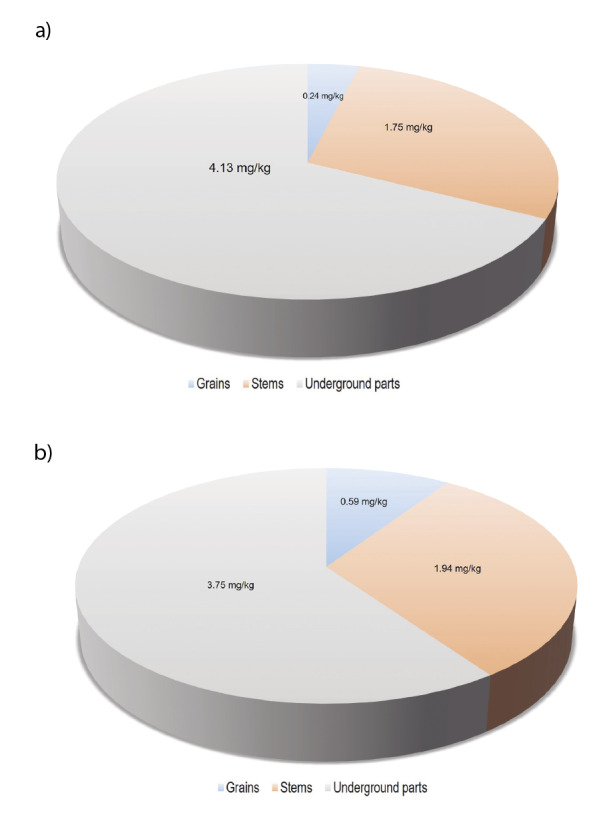
Distribution of Cd content in durum wheat organs of: a) cultivars with alleles associated with low and b) high Cd content

In addition to grain, stem Cd mass fractions were determined (Fig. 2b). The addition of Cd (8 mg/kg) to the soil mixture increased the stem Cd mass fraction in almost all cultivars used in pot experiment. Moreover, among control samples, Gap cultivar had the highest stem Cd mass fraction, while Tüten 2002 cultivar had the lowest (Fig. 2b and Table S2). Durum wheat cultivars with low Cd mass fraction in their stems can be beneficial feed sources especially for small ruminants that graze wheat stems and leaves after grain harvest under rainfed conditions in Turkey. Svevo cultivar also had the highest stem Cd mass fraction in addition to its high grain Cd accumulation (Fig. 2b). The results of a two-way ANOVA show that there were significant differences (p<0.01) in mass fractions of all elements determined in stem for cultivar and cultivar × treatment interaction (Table S2). On the other hand, Cd mass fractions in underground parts (roots and stubble) of cultivars grown in the soil with the addition of 8 mg/kg Cd were also determined and the results showed that most of the added Cd was accumulated by the plants (Fig. 2c). While Amanos-97 cultivar had the lowest Cd mass fraction in each organ in general, Ege-88 cultivar had the highest Cd mass fraction in the underground parts in particular (Fig 2c). Considering Cd distribution in plant organs, most of the Cd was found in the underground parts, in which cultivars with the alleles associated with low Cd content had 4.13 mg/kg *i.e*. 67% total Cd (Fig. 3a), whereas those containing alleles associated with high Cd content had 3.75 mg/kg, *i.e.* 60% of the total Cd (Fig. 3b).

### Correlation and multivariate analyses of Cd mass fraction in the grain

In order to understand the relationships among elements, correlation analysis was performed (Table 3). There was a negative correlation between the grain Cd and Cu (*r*=-0.76, p<0.01) and Mn (*r*=-0.56, p<0.01) in the control samples. Grain Cd was also negatively correlated with Mg (*r*=-0.55, p<0.01) in the grain samples grown in soil with added Cd (Table 3). An opposite finding was reported by Perrier *et al.* (*22*) that grain Cd was positively correlated with Mn (*r*=0.61, p<0.01) and Mg (*r*= 0.38, p<0.05). In addition to these, there was a non-significant correlation between the grain Cd and Cu (*22*). Liu *et al.* (*23*) also studied correlations between Cd and mineral nutrients in parts of roots and leaves in rice and they reported that Cd^2+^ was generally correlated with Fe^3+^, Mn^2+^, Cu^2+^ and Mg^2+^. Jalil *et al.* (*24*) conducted a similar study of durum wheat with different Cd mass fraction added to nutrient solution and they reported that for all of them, the mass fractions of Mn, Zn, Cu and Fe were not affected significantly but Cd additions to the solution depressed the uptake of Zn and Mn. A similar negative interaction between Cd and Mn was also found in this study.

**Table 3:** Correlations between Cd and other elements in the grain of control samples and samples grown in soil with the addition of *w*(Cd)=8 mg/kg

Treatment	P	K	Ca	Mg	Fe	Zn	Cu	Mn
Control	-0.32	-0.08	-0.28	0.15	-0.26	-0.21	-0.76*	-0.56*
*w*(Cd)=8 mg/kg	-0.27	0.36	0.27	-0.55*	-0.35	-0.19	-0.48	-0.31

*p<0.01

**Fig. 4:**
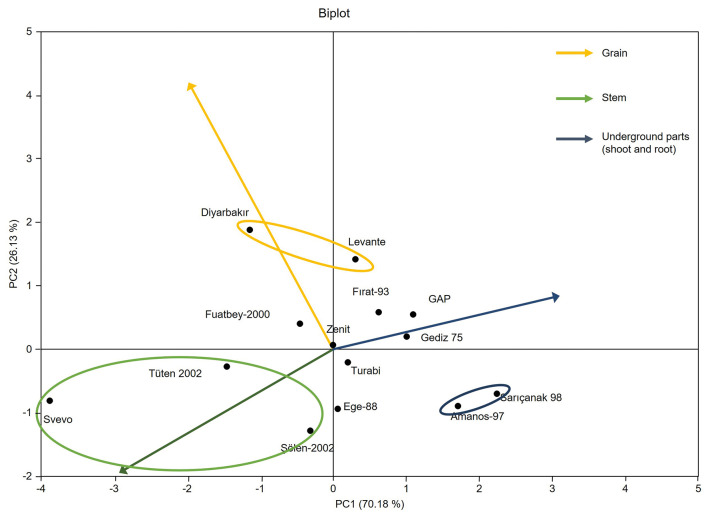
Bi-plot obtained with principal component analysis (PCA) of Cd mass fractions in plant organs of different durum wheat cultivars

Additionally, principal component analysis (PCA) was performed to determine the relationships between genotypes and plant organs (Fig. 4). PCA showed that the first two components (PC1 and PC2) accounted for 96.31% of the total variance. PC1 explained 70.18% variance, while PC2 elucidated 26.13% of the total variance (Fig. 4). Moreover, contribution of each plant organ to the PC1 and PC2 shows that Cd in the underground parts of plant (43.51) was major contributor to PC1, whereas Cd in the grain (79.72) mainly contributed to PC2 (Table 4). Vergine *et al.* (*21*) similarly performed PCA for determination of Cd mass fraction in durum wheat and they reported that roots and kernels contributed to PC1 and grains mostly contributed to PC2. As a result of biplot visualization, different groups were revealed for each plant organ such as underground part (shoots and roots), stem and grain (Fig. 4). Each circle represents different group of Cd mass fractions in each plant organ in the bi-plot graph. The Diyarbakır and Levante cultivars, marked with yellow color, accumulate the highest mass fractions of Cd in the grain. The second group marked with green consists of Svevo and Tüten 2002 cultivars, which had the highest mass fractions of Cd in the stem. The third group marked with blue color comprises Amanos-97 and Sarıçanak 98 cultivars, which accumulate high mass fractions of Cd in the root. All other cultivars, Fırat-93, GAP, Fuatbey-2000, Zenit, Gediz 75, Turabi and Ege-88, had lower Cd mass fractions in all plant organs (Fig. 4). Svevo cultivars accumulated the highest mass fraction of Cd in both grain and stem, while Amanos-97 cultivar had the lowest mass fraction of Cd in the stem and grain. At this point, difference in the root to grain translocation of Cd among durum wheat genotypes is very important to develop new cultivars that can be grown in Cd-contaminated soils. If this translocation is weak or root sequestrates the Cd efficiently, grain Cd content will be low (*25*, *26*). In addition to this, partitioning of Cd among plant organs is the second important strategy for low Cd mass fraction in the grain. Perrier *et al.* (*22*) highlighted that growing long-stemmed cultivars may have advantages since lower mass fractions of Cd are moved to plant organs such as stem, leaves, bracts, rachis and grains. Arduini *et al.* (*27*) found that partitioning to shoots and grains with increasing Cd supply was markedly higher in Svevo cultivar. They also reported that high Cd content in grains of Svevo cultivar may be related to the high allocation of biomass in roots during vegetative growth stage coupled with high post-heading dry matter accumulation and root to grain re-mobilization. Higher accumulation of elements from the soil in the plant is a desired trait to obtain higher yield and quality; therefore, breeding studies have focused on improving yield components to increase crop yield (*28*). Due to these concerns, modern wheat varieties tend to accumulate elements such as Cd in the grain (*21*, *22*, *28*). However, since high Cd content in the grain is not a desirable trait, cultivars with alleles associated with low Cd content and high yield should be given first priority in durum wheat breeding.

**Table 4:** The results of principal component analysis (PCA) of plant organ (underground parts, stems and grains) contribution to Cd accumulation in durum wheat cultivars

Plant organ	PC1	PC2
Underground parts (root and stubble)	**43.51**	3.19
Stem	**38.82**	17.10
Grain	17.68	**79.72**

## CONCLUSIONS

In a nutshell, Cd is released into the environment in many ways, including the use of intensive phosphate fertilizers, sewage sludge and fossil fuel combustion in addition to natural Cd sources, and therefore Cd contamination of the soils has increased worldwide. In recent years, lower accumulation of Cd has been a breeding priority in addition to other quality traits especially in Cd-contaminated soils, and many wheat varieties have been developed with marker-assisted breeding. In this study, molecular analysis showed that 24 durum wheat cultivars, one emmer wheat and four wild emmer genotypes accumulated high mass fractions of Cd, while 68 genotypes had the allele associated with low Cd accumulation. Moreover, these molecular findings were supported by elemental analyses performed after pot experiment using a small set of 14 cultivars. In conclusion, since chemical or elemental analyses are expensive and time consuming for selection of genotypes with low levels of Cd, marker-assisted studies can be effectively used for both selection and introgression of *Cdu1* alleles to adapted common durum wheat cultivars with low grain Cd content.

## Figures and Tables

**Table S1:** Accumulation of cadmium and other elements in durum wheat grain (control samples and samples grown in soil with the addition of *w*(Cd)=8 mg/kg (dose))

Cultivar	*w*/(mg/kg)																	
Cd		P		K		Ca		Mg		Fe		Zn		Cu		Mn	
Control	Dose	Control	Dose	Control	Dose	Control	Dose	Control	Dose	Control	Dose	Control	Dose	Control	Dose	Control	Dose
Ege-88	0.18	0.30	2175.10	2394.67	8136.33	5296.00	606.80	358.10	812.30	854.33	32.89	20.44	35.89	27.81	3.94	2.38	18.33	19.58
Amanos-97	0.19	0.12	2684.67	2429.67	5246.00	5020.67	429.60	408.93	869.00	984.63	21.51	39.84	37.55	41.47	3.23	10.75	28.45	30.57
Sarıçanak 98	0.15	0.13	2262.00	2408.67	5123.67	4813.67	341.77	346.87	804.40	984.63	14.62	24.54	22.30	27.09	2.27	3.21	17.49	23.40
Şölen 2002	0.13	0.24	2275.67	2240.00	6685.67	5907.33	294.07	379.00	869.83	946.80	13.75	32.75	30.91	29.68	3.84	2.99	19.22	24.68
Turabi	0.10	0.40	2097.00	1936.00	5926.33	6598.67	481.53	525.33	865.10	841.17	31.54	27.03	32.27	25.75	4.92	4.43	23.19	29.04
Svevo	0.27	0.65	2673.67	2420.00	6119.33	5406.33	416.33	492.03	912.30	904.00	19.76	13.15	28.30	26.11	2.92	2.40	21.37	23.25
Zenit	0.21	0.46	2728.67	2448.00	7261.67	9801.67	428.37	551.10	989.83	728.20	15.01	28.32	33.27	26.47	3.23	2.60	14.94	20.51
Fırat-93	0.35	0.53	2298.00	2602.00	7208.67	7093.00	429.17	316.97	965.43	949.33	18.73	27.60	34.51	31.19	2.48	3.65	16.46	19.87
Fuatbey 2000	0.32	0.59	2371.67	2507.00	6500.67	7341.33	336.83	338.97	877.50	877.47	16.46	20.61	28.57	29.34	3.29	3.12	16.59	21.09
GAP	0.28	0.47	2081.33	2535.33	4668.33	5177.33	307.67	371.13	812.50	870.07	20.05	24.14	24.38	25.45	2.08	2.49	17.46	21.70
Gediz 75	0.36	0.42	2113.67	2161.67	6691.00	5862.67	348.40	502.87	811.43	771.17	17.46	30.60	32.71	21.57	2.38	2.84	15.39	18.07
Tüten 2002	0.37	0.55	1997.67	1771.33	5074.00	5643.33	246.97	382.43	844.33	862.70	17.09	25.75	25.98	28.81	1.94	1.98	18.37	25.37
Diyarbakır	0.38	0.91	1915.67	2074.33	4742.67	5819.33	429.30	457.17	974.90	768.17	20.41	23.74	29.62	32.44	2.00	2.36	14.61	25.16
Levante	0.36	0.7	2169.67	2033.33	7278.00	8852.33	418.27	476.07	803.03	845.93	22.39	35.95	26.05	26.56	2.21	3.62	13.30	16.63
F (C)	97.96**		7.85**		68.21**		12.12**		5.44**		21.76**		20.36**		62.28**		32.36**	
F (C × T)	28.12**		2.13*		21.65**		7.89**		9.20**		26.92**		8.45**		44.85**		3.41**	
LSD (0.05)	0.11		250.91		868.89		80.90		86.33		6.23		3.74		1.40		2.27	

C=cultivar, T=treatment; *p<0.05, **p<0.01

**Table S2:** Accumulation of cadmium and other elements in durum wheat stem (control samples and samples grown in soil with the addition of *w*(Cd)=8 mg/kg (dose))

Cultivar	*w*/(mg/kg)																	
Cd		P		K		Ca		Mg		Fe		Zn		Cu		Mn	
Control	Dose	Control	Dose	Control	Dose	Control	Dose	Control	Dose	Control	Dose	Control	Dose	Control	Dose	Control	Dose
Ege-88	0.66	2.34	1124.33	1117.10	15320.00	13540.00	4072.00	3974.33	1197.67	913.93	124.47	154.54	99.22	111.37	1.23	0.94	20.97	24.31
Amanos-97	0.70	1.20	1046.00	1533.33	12613.33	12830.00	4600.33	3922.33	896.10	977.90	100.15	148.70	53.96	57.30	0.70	3.69	37.25	32.62
Sarıçanak 98	0.57	1.20	749.43	727.97	12550.00	12370.00	3866.67	4234.00	1290.00	1272.33	134.77	142.97	99.24	70.62	3.73	3.52	24.55	27.96
Şölen 2002	0.50	2.23	302.97	251.70	13156.67	13443.33	2846.67	3722.00	865.10	987.17	84.83	108.72	65.59	72.12	2.81	3.11	29.04	46.75
Turabi	0.57	1.78	273.90	302.13	14536.67	12846.33	3484.00	3454.00	806.80	875.93	88.04	199.20	61.35	47.81	2.13	1.28	23.73	40.12
Svevo	0.56	3.50	529.67	437.97	14973.33	14560.00	3800.00	4665.67	999.73	1041.77	129.85	120.93	81.02	97.14	1.06	3.13	34.35	53.43
Zenit	0.63	1.67	498.70	494.70	11513.33	14066.67	3227.00	3572.00	774.67	633.70	103.84	109.83	166.09	42.96	3.97	4.81	17.55	26.66
Fırat-93	0.70	1.64	247.00	308.30	14190.00	13906.67	3367.00	4838.33	624.17	834.80	140.30	150.07	53.91	71.39	3.68	4.06	16.58	33.41
Fuatbey 2000	0.61	2.07	330.30	500.73	17786.67	18696.67	3716.00	3473.33	760.63	705.87	104.54	138.36	49.40	68.49	1.21	1.86	16.90	30.52
GAP	0.82	1.33	193.17	243.73	15181.00	16236.67	3789.33	4537.67	915.90	911.60	98.73	181.43	81.45	79.89	3.89	5.63	12.51	35.60
Gediz 75	0.62	1.55	342.00	601.27	13330.00	15946.67	3187.00	3927.00	875.00	1048.67	135.00	189.73	48.79	70.97	1.38	2.07	14.73	33.68
Tüten 2002	0.48	2.61	360.43	643.97	12563.33	18936.67	3728.33	6710.67	804.73	1198.67	146.10	159.84	61.39	86.37	0.23	3.15	27.42	38.33
Diyarbakır	0.60	1.71	288.07	472.23	13506.67	15360.00	3796.67	4892.33	875.07	1121.30	127.23	151.24	71.77	92.44	0.62	2.89	17.04	35.24
Levante	0.65	1.36	277.40	366.53	15548.00	17310.00	3466.67	4367.33	640.53	878.73	150.07	147.10	51.80	71.78	2.91	4.30	16.18	25.70
F (C)	36.06**		254.46**		48.18**		275.06**		141.40**		19.51**		106.87**		69.26**		663.40**	
F (C × T)	48.83**		15.22**		19.72**		16.56**		10.36**		17.25**		126.73**		17.81**		76.58**	
LSD (0.05)	0.43		146.58		1382.00		609.37		124.18		22.97		23.46		0.81		22.97	

C=cultivar, T=treatment; *p<0.05, **p<0.01
